# Targeting Reactive Oxygen Species for Diagnosis of Various Diseases

**DOI:** 10.3390/jfb15120378

**Published:** 2024-12-15

**Authors:** Moung Young Lee, Donguk Lee, Dayun Choi, Kye S. Kim, Peter M. Kang

**Affiliations:** 1 Cardiovascular Institute, Beth Israel Deaconess Medical Center, Harvard Medical School, Boston, MA 02115, USA; 2 Centers for Research in ICT based Infectious Diseases, Jeonbuk National University, Jeonju 561-756, Republic of Korea; 3 Massachusetts College of Pharmacy and Health Sciences, Boston, MA 02115, USA

**Keywords:** reactive oxygen species, diagnostic imaging, targeted imaging agents, theragnostic

## Abstract

Reactive oxygen species (ROS) are generated predominantly during cellular respiration and play a significant role in signaling within the cell and between cells. However, excessive accumulation of ROS can lead to cellular dysfunction, disease progression, and apoptosis that can lead to organ dysfunction. To overcome the short half-life of ROS and the relatively small amount produced, various imaging methods have been developed, using both endogenous and exogenous means to monitor ROS in disease settings. In this review, we discuss the molecular mechanisms underlying ROS production and explore the methods and materials that could be used to detect ROS overproduction, including iron-based materials, ROS-responsive chemical bond containing polymers, and ROS-responsive molecule containing biomaterials. We also discuss various imaging and imaging techniques that could be used to target and detect ROS overproduction. We discuss the ROS imaging potentials of established clinical imaging methods, such as magnetic resonance imaging (MRI), sonographic imaging, and fluorescence imaging. ROS imaging potentials of other imaging methods, such as photoacoustic imaging (PAI) and Raman imaging (RI) that are currently in preclinical stage are also discussed. Finally, this paper focuses on various diseases that are associated with ROS overproduction, and the current and the future clinical applications of ROS-targeted imaging. While the most widely used clinical condition is cardiovascular diseases, its potential extends into non-cardiovascular clinical conditions, such as neurovascular, neurodegenerative, and other ROS-associated conditions, such as cancers, skin aging, acute kidney injury, and inflammatory arthritis.

## 1. Introduction

ROS play essential roles in numerous physiological processes, including cellular growth, differentiation, proliferation, signaling, and senescence [1,2,3]. However, excessive ROS accumulation could also result in pathological disruptions through cellular dysfunctions, impairing ion pumps and inducing apoptosis [3,4]. Consequently, many researchers have developed methods to detect and mitigate ROS overproduction. For the effective detection of ROS, imaging modalities that are specific to ROS are required. However, currently available clinical applications lack modalities that specifically monitor ROS overproduction, since ROS are very short-lived and exist in very low levels. To address these issues, synthesized materials are combined with contrast agents, which enhance drug delivery and amplify signals in regions with elevated ROS levels [5,6,7]. Iron-based materials, for instance, utilize redox-catalytic processes to reduce ROS levels [8]. Additionally, hydrogen peroxide (H_2_O_2_)-targeted biomaterials and selenium are frequently used for drug delivery through chemical interactions with ROS [9,10]. In this paper, we review materials designed to detect ROS and explore the imaging modalities for ROS monitoring. Additionally, we examine the clinical applications of ROS-targeted imaging in various diseases.

## 2. Molecular Mechanism of ROS Production

The molecular mechanisms that are involved with ROS production are complex. This current review is intended to give a brief overview of these mechanisms. There are a number of review articles written on this topic [1,2,11], and we encourage the readers to refer to these papers for more details.

ROS play vital roles in cellular functions, such as respiration, growth, differentiation, signaling, and senescence. However, excessive ROS accumulation disrupts these processes and can cause pathological effects. Oxidative stress occurs when the production of ROS, such as super oxide, H_2_O_2_, hydroxyl radical, and alkoxy/peroxy-radicals, exceeds the capacity of the antioxidant systems [3,4]. These chemically unstable oxygen free radicals or nitrous free radicals metabolize the protein, lipid, and other biomolecules in the body.

**Figure 1:**
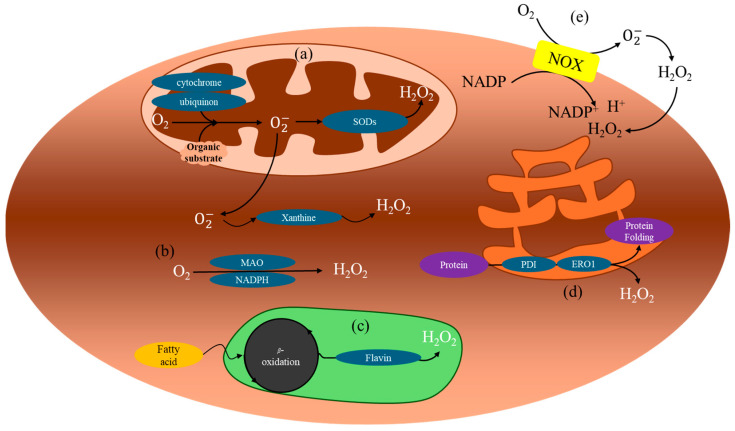
Molecular mechanisms of H_2_O_2_ production. (**a**) H_2_O_2_ is generated during cellular respiration in the mitochondria. This process generates superoxide anion through ubiquinone and cytochrome. Superoxide anion is rapidly converted to H_2_O_2_ in the mitochondria by superoxide dismutases (SODs) and in the cytosol by xanthine oxidase. (**b**) H_2_O_2_ can also form through the action of NADPH oxidase and monoamine oxidase. (**c**) In peroxisomes, H_2_O_2_ is generated during fatty acid β-oxidation. (**d**) Proteins generate H_2_O_2_ by endoplasmic reticulum oxireductin 1 (ERO1) and protein disulfide isomerase (PDI) in the endoplasmic reticulum. An increase in proteins under physiological conditions promotes oxidative folding, resulting in electron transfer between PDI and ERO1, generating H_2_O_2_. (**e**) NADPH oxidase (NOX) interacts with O_2_ in the extracellular space, producing superoxide anion, which is then converted to H_2_O_2_.

Endogenously, ROS are primarily generated during mitochondrial respiration (Figure 1). In this process, oxidase enzymes receive electrons from organic substrates via membrane carriers like ubiquinone and cytochrome c, leading to the formation of superoxide radicals. These radicals generated in the mitochondria are rapidly converted to H_2_O_2_ by superoxide dismutases (SODs) or xanthine oxidase. Additionally, H_2_O_2_ can be generated without superoxide intermediates via nicotinamide adenine dinucleotide phosphate (NADPH) oxidase and monoamine oxidase. Other sources of ROS include fatty acid β-oxidation, flavin oxidase activity in peroxisomes, and protein oxidation in the endoplasmic reticulum. Externally, environmental factors, such as cigarette smoke and ultraviolet (UV) light, contribute to ROS production [4].

The half-life of superoxide is very short and imaging it has been difficult. Among ROS, H_2_O_2_ is the most abundant due to its relatively long biological half-life and stability. It is highly diffusible within and between cells, facilitated by both passive diffusion and specialized aquaporin channels [12,13]. While H_2_O_2_ plays an essential role at physiological levels by regulating cellular signaling, excessive production can lead to oxidative stress, causing tissue damage through the release of pro-inflammatory cytokines and activation of apoptosis [14,15,16,17]. The body maintains redox homeostasis through enzymatic and non-enzymatic defense mechanisms. Under normal conditions, H_2_O_2_ modulates cellular functions by phosphorylating kinases and acting as an oxidant signal to regulate the biological responses. However, H_2_O_2_ can interact with Fe^2+^ and Cu^+^ ions to produce hydroxyl radicals, which are highly reactive and damaging to cells (via the Haber–Weiss reaction) [18]. SODs and catalases play crucial roles in preventing excessive ROS accumulation by catalyzing the conversion of superoxide radicals into H_2_O_2_ and subsequently into water and oxygen [4]. An imbalance of this redox homeostasis could lead to the modifications of membrane lipids, proteins, and nucleic acids, which could ultimately lead to various diseases. Cardiovascular and central nerve systems are especially vulnerable to oxidative stress due to high metabolic rates and energetic demands.

## 3. Materials Used for Diagnostic and Therapeutic Targeting of ROS

Currently, no Food and Drug Administration (FDA)-approved materials exist for detecting oxidative stress in clinical settings. However, methods for detecting oxidative stress are widely applied in experimental research settings, typically using fluorogenic and non-fluorogenic approaches. These materials are often synthesized with multifunctional properties, including targeting, therapeutic, and imaging capabilities.

### 3.1. Iron-Based Materials

Although some iron oxide particles are used in clinical research, they are not currently approved for patient use in ROS imaging [19]. Iron oxide nanoparticles, a class of ferromagnetic materials, have applications in biomedical and bioengineering fields [8]. Due to iron’s variable oxidation states, Fe^2+^ and Fe^3+^, and redox-catalytic activity, it plays an important role in ROS-related chemistry [20]. Engineered iron structures, such as nano-iron metals and nano-iron oxides, are utilized in various medical fields, including intravenous iron therapies, iron supplements, MRI contrast agents, drug delivery, tissue engineering, and hyperthermia [19,21,22]. A deeper understanding of the ROS-related activities of these iron-based materials is essential to address safety concerns associated with increased biological exposure to nanostructures [20]. For example, the use of superparamagnetic iron oxide (SPIO) as an imaging contrast in human patients has been withdrawn by FDA for safety concerns although it remains approved for use in anemic patients who are refractory to conventional iron pills.

Ferrocene-based materials demonstrate ROS-responsive redox activity. Hydrophobic ferrocene, when copolymerized with hydrophilic monomers, forms amphiphilic block copolymers that self-assemble into nanocarriers. Upon oxidation, the hydrophobic components become hydrophilic, triggering polymer disassembly and controlled drug release. ROS-responsive nanocarriers containing ferrocene blocks and carboxyl groups have been developed for drug delivery [23]. However, low yield, poor aqueous solubility, and challenges in controlling physicochemical properties are limiting factors that must be addressed to enhance therapeutic efficacy. One advantage of block copolymers is the ability to fine-tune nanoparticle properties by adjusting block composition and length [24].

### 3.2. ROS-Responsive Chemical Bond Containing Polymers

Another strategy is to design biomaterial that are ROS-responsive due to specific chemical bonds within the polymer backbone that are cleaved in the presence of ROS. This allows the release of imaging agents that are encapsulated in the polymer.

#### 3.2.1. Peroxalate Ester-Based Polymers

Peroxalate ester-based polymers offer a promising approach for ROS-targeted applications [25,26,27]. These polymers react with H_2_O_2_, leading to alcohol and CO_2_ formation, which can trigger drug release from nanoparticles [9,28]. Peroxalate polymers can also function as contrast agents in H_2_O_2_-associated diseases by generating chemiluminescence in response to ROS [9,28]. The dioxetane dione intermediate produced by these nanoparticles enables the emission of high-wavelength light, which can be used for fluorescence imaging. This unique feature allows fluorescent dyes embedded in the nanoparticles to be excited, enhancing imaging performance [29]. These peroxalate ester-based polymers have shown potential application in both diagnostic and therapeutic uses in various ROS-associated conditions [26,27].

#### 3.2.2. Boron-Containing Polymers

Phenylboronic acid are very sensitive to ROS due to the boron-carbon bond, and undergo rapid oxidative degradation when exposed to ROS [30,31]. Boronic ester bonds exhibit degradation even as low as 50 μM of H_2_O_2_. Hydrogel drug delivery system that incorporated boronic ester bond material has been also reported for treatment of myocardial ischemia reperfusion injury (MIRI) [32,33]. We speculate that encapsulating imaging agent using this strategy is feasible.

#### 3.2.3. Poly-Thioketal Polymers

Similar to boronic esters, thioketal linkages are sensitive to ROS, such as H_2_O_2_, leading to the oxidation into ketones and organic thiols [34]. This strategy has been used for ROS-responsive drug delivery system in various disease conditions [35,36].

#### 3.2.4. Poly-Proline-Containing Polymers

Poly-proline has been shown to be responsive to ROS due to its pyrrolidine rings [37]. In the presence of ROS, the proline residue undergoes oxidation, leading to degradation of the polymer backbone [38]. This causes the release of encapsulated imaging agent. In tissue engineering application, poly-proline has been used to generate the ROS-responsive scaffolds [39,40].

### 3.3. ROS-Responsive Molecule Containing Biomaterials

The biomaterials in this group contains molecules that undergo solubility changes in response to ROS. In the presence of ROS, they usually transform from hydrophobic to hydrophilic states. This mechanism results in enhanced water solubility and allows the release of encapsulated agents.

#### 3.3.1. Selenium-Containing Biomaterials

Selenium acts as an antioxidant through specific selenoproteins, which directly protect cells from oxidative stress [10]. One key enzyme, thioredoxin reductase, plays a critical role in regenerating low molecular weight antioxidants, highlighting the selenium’s importance in redox regulation. However, the effectiveness of selenium varies depending on the dosage and the chemical form used. For example, redox-inactive forms, such as selenomethionine, have shown limited success in clinical studies [41].

While selenium can mitigate oxidative stress, excessive levels may paradoxically increase ROS production leading to oxidative damage [42]. Interestingly, selenium exhibits selective toxicity by inducing cell death in tumor cells at concentrations that are non-toxic to normal cells [43,44]. In ROS-rich environments, hydrophobic selenium groups convert to hydrophilic selenosulfones, destabilizing nanoparticles or micelles, and triggering drug release.

#### 3.3.2. Sulfur-Containing Biomaterials

Because of the diverse oxidation states of sulfur, sulfur-containing biomaterials could undergo significant changes upon exposure to ROS. When oxidized, sulfide-containing polymers form sulfoxides or sulfones, and increase their hydrophilicity. This change leads to polymer swelling, disassembly, and eventual drug release [45]. Poly-propylene sulfide (PPS) is widely used sulfur-containing ROS-responsive functional group. PPS-based nanoparticles degrade rapidly in the presence of H_2_O_2_, which allow encapsulated agents to be released [46]. In addition, poly (L-methionine) has been shown to be ROS-responsive through the oxidation of its sulfur-containing methionine residues to sulfoxides and sulfones. [47,48]. This oxidation causes an alteration in hydrophobicity that causes structural changes, which promotes the release of encapsulated agents.

#### 3.3.3. Tellurium-Containing Biomaterials

Tellurium is another molecule that has high sensitivity to ROS due to its chemical properties, making tellurium-containing polymers extremely responsive to ROS. This property is important in an environment where there is a low level of ROS [49,50]. Tellurium-containing biomaterials also exhibit significant solubility changes under oxidative conditions [50], which allows the effective release of the encapsulated agent. The potential diagnostic and therapeutic applications are being explored, but it is still in the research stage [51].

#### 3.3.4. Diselenide, Disulfide, and Ditelluride Bond-Containing Biomaterials

More recent studies demonstrate that diselenide (-Se-Se-), disulfide (-S-S-), and ditelluride (-Te-Te-) bond-containing biomaterials show improved ROS-responsive properties [52,53,54]. These bonds are sensitive in the presence of ROS and degraded, which allow the release of encapsulated agents. Diselenide bonds respond rapidly to ROS, and have been explored in application for cardiovascular and neurodegenerative diseases [53,55]. The Se-Se bond within selenium compounds breaks down under oxidative stress, resulting in the formation of selenic acid and the release of encapsulated drugs during the depolymerization process [10]. Disulfide bonds have a higher bond dissociation energy than diselenide bonds, which makes them ideal for delivery systems [54,56]. Ditellurium is the most sensitive to ROS and can respond rapidly. There have been more studies performed to explore its potential application in various disease states that are associated with ROS [52,57].

## 4. Contrast Agents for ROS Imaging

Contrast agents play an essential role in enhancing the sensitivity and accuracy of various imaging modalities, particularly for visualizing ROS production and oxidative stress. These agents improve the signal-to-noise ratio (SNR), increase the tissue contrast-to-noise ratio (CNR), and help to detect and to diagnose pathophysiological changes.

### 4.1. Gadolinium-Based Contrast Agents (GBCAs)

GBCAs are among the most widely used contrast agent in MRI, approved by the FDA. Gadolinium has paramagnetic property that interacts with water molecules, shortening their T1 relaxation time and enhancing signal intensity on T1-weighted MRI images [58]. These agents help visualize infection, inflammation, and tumors in various organs on MRI and MRA. GBCAs have (1) high tissue contrast, which improves lesion detection, (2) multi-contrast capability, which is useful for different MRI sequences and acquisition timing, and (3) ability to perform functional imaging that enables MRI to monitor functional changes. However, the limitations are rare adverse reactions and nephrotoxicity, such as nephrogenic sclerosis, and gadolinium retention, especially in patients with kidney dysfunction. Thus, screening patients before administration of GBCA in non-stroke patients are important and limits its use [5,58].

### 4.2. Indocyanine Green (ICG)

ICG is an FDA-approved contrast agent widely used in fluorescence imaging and other diagnostic applications. It emits near-infrared light (NIR) with a peak at 805–810 nm, providing a high signal-to-noise ratio for biomedical applications [6,59]. ICG is particularly useful for monitoring ROS-related therapeutic effects in real time. The advantages of ICG are a high signal-to-noise ratio and NIR range, which allow better tissue penetration compared to visible light. Some of the limitations are instability, rapid clearance, and relatively non-targeted delivery reducing specificity for certain tissues. To overcome these limitations, researchers have developed polymeric platforms to encapsulate ICG, which can improve stability and reach the target areas with higher specificity [6,59].

### 4.3. Microbubbles

Microbubbles are widely used as contrast agents for US imaging due to their unique ability to enhance acoustic scattering [7,60,61]. With a size comparable to red blood cells (~10 µm), microbubbles are particularly effective in visualizing microvascular structures and monitoring vascular conditions [62]. Their gas-core structure enhances ultrasound signals. This allows their applications in drug delivery [62,63] and photoacoustic imaging (PAI) with improved contrast through vaporizing nanodroplets consisting of perfluorocarbon and gold nanorods [64,65]. The advantages of microbubble are its (1) high acoustic backscatter that could enhance ultrasound signals, (2) ability to perform microvascular imaging, and (3) versatility (used in drug delivery and improve PAI). However, its limitations are due to its short circulation time and limited tissue penetration.

## 5. Imaging Modalities for ROS

Imaging modalities play a crucial role in diagnosing and monitoring oxidative stress and ROS-related conditions. However, detecting ROS is challenging due to their short half-life and variable productions and concentrations across different cellular compartments. While real-time imaging of ROS is desirable, achieving precise spatial and temporal resolution remains difficult with existing technologies. Researchers are exploring targeted materials for more efficient ROS imaging and reducing the materials needed for diagnosis. These targeted and ROS-sensitive materials are often combined with imaging contrast agents, enabling the visualization of ROS distribution and therapeutic effects. Below describes the key imaging modalities used to monitor ROS production and oxidative stress.

### 5.1. Magnetic Resonance Imaging (MRI) (Figure 2a)

MRI is a non-invasive, widely used imaging modality that visualizes internal structures using nuclear magnetic resonance principles [66]. MRI primarily relies on hydrogen nuclei in the body aligning with a strong magnetic field. When a radiofrequency pulse disrupts this alignment, hydrogen nuclei emit signals as they return to their equilibrium state, which are detected by receiver coils and reconstructed into images via Fourier transformation [67,68].

MRI has applications in both clinical and preclinical research for monitoring ROS-related changes. Through magnetic resonance properties, MRI can visualize oxidative stress by detecting signal changes in regions of elevated ROS production. For example, H_2_O_2_ accumulation will be shown with a shortening of T1 relaxation. While MRI offers excellent spatial resolution and depth perception through cross-sectional imaging, it is limited by its high cost, availability, and lack of real-time imaging. Only those who are compatible with MRI can be imaged after thorough screening and interrogation. It is often used in MR-guided surgeries in a limited number of sites, but MRI cannot capture the dynamic real-time distribution of ROS at precise moments. However, in chronic inflammation and neoplastic processes, accumulated ROS can be imaged and can be useful in the diagnosis, treatment and prevention.

**Figure 2:**
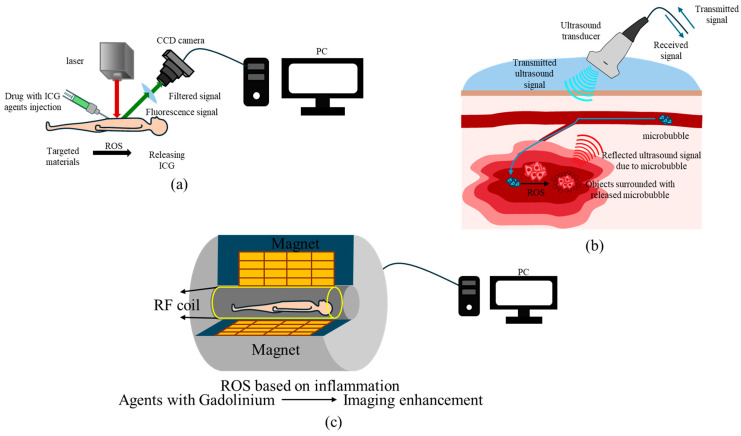
Clinical imaging modalities to detect ROS. (**a**) Fluorescence imaging using drug/contrast agent delivery system. When ROS-targeting materials are injected into the body, they be released at the site of ROS overproduction, which allows detection using fluorescence imaging. (**b**) Ultrasound imaging using microbubble-based drug delivery. When directed to ROS-overproducing targets, the microbubble structure is disrupted. This enhances the ultrasound signal, which can be detected using an ultrasound transducer. (**c**) MRI imaging using Gadolinium-based materials. Gadolinium is released near the target, amplifying the T1-weighted signal.

### 5.2. Sonography (Figure 2b)

Sonography takes advantage of ultrasound (US) and is widely used imaging modality that relies on sound waves with various frequencies to generate images of tissues and diseases based on their acoustic properties. When ultrasound waves pass through tissues, echoes are generated due to differences in tissue properties and the residual signals are returned to the ultrasound probe, which are reconstructed into images [69].

While ultrasound is cost-effective and can detect deeper tissues, it struggles to penetrate structures obscured by bone or gas. To improve its functionality, contrast agents, such as microbubbles, are used to increase the high acoustic scattering properties. Doppler ultrasound can measure blood flow, but its capability to detect ROS directly remains limited. Sonography can require a high level of training and expertise that often result in highly user-dependent accuracy.

### 5.3. Fluorescence Imaging (Figure 2c)

Fluorescence imaging uses light to visualize biological structures by exciting fluorescent molecules and detecting the emitted signals with high-sensitivity cameras [70,71]. This technique often employs near-infrared (NIR) light to excite contrast agents or fluorophores, which are widely used in ROS-targeted research. Fluorescence imaging is advantageous for the real-time visualization of ROS distribution and monitoring therapeutic effects. However, it is limited by poor tissue penetration, as light cannot reach deep tissues. As a result, fluorescence imaging is effective for surface-level or near-surface monitoring. Its relatively inexpensive cost and wide availability make it useful during biopsied or ex vivo samples for ROS in clinical setting.

### 5.4. Photoacoustic Imaging (PAI) (Figure 3a)

PAI combines optical and ultrasound imaging by using light pulses to generate acoustic waves through thermal expansion in tissues [72]. When tissues absorb pulsed light, they emit acoustic signals, which are detected by ultrasound receivers to create high-resolution images. PAI combines the chemical specificity of optical imaging with the deeper penetration capability of ultrasound.

**Figure 3:**
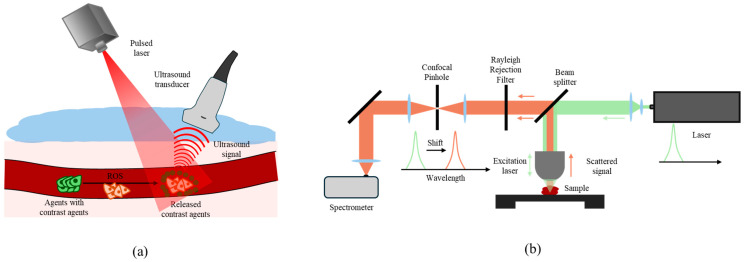
Preclinical imaging modalities to detect ROS. (**a**) Photoacoustic imaging. When contrast agent-loaded materials reach ROS overproduced regions, they release drugs and contrast agents. The contrast agents amplify the ultrasound signal upon pulsed laser irradiation. (**b**) Raman imaging. When the laser is applied to the sample, the sample molecules vibrate, causing a shift in the light wavelength. Rayleigh signals are filtered out. The remaining signals are organized by wavelength to reconstruct images.

PAI provides real-time imaging and can be enhanced with nanoparticles or fluorescent dyes to increase sensitivity to ROS. By combining structural imaging from ultrasound and the functional imaging aspect of PAI, researchers can visualize ROS distribution and therapeutic responses. This modality offers a promising approach for tracking ROS production dynamically in disease settings.

### 5.5. Raman Imaging (Figure 3b)

Raman imaging, based on the Raman effect, detects molecular vibrations by analyzing the inelastic scattering of incident light interacting with target molecules [73,74]. When a monochromatic laser interacts with a sample, most of the scattered light retains the same frequency, but a small fraction shows a frequency shift, producing Raman spectra [75,76,77]. This shift reveals molecular-level information, making Raman spectroscopy useful for studying DNA, proteins, and oxidative stress markers.

Raman imaging offers a high spatial resolution, low background noise, and good photostability, making it useful for microscopy and molecular analysis. However, the signals generated through Raman scattering are inherently weak, limiting its application in biomedical imaging. Enhancing Raman signal strength through advanced contrast agents or combined modalities is an area of ongoing research. Its selection in areas of application such as superficial organs might be an approach for successful implementation.

## 6. Applications of ROS-Targeted Imaging in Cardiovascular Diseases

The overproduction of ROS is intrinsically linked to a variety of cardiovascular pathologies, including myocardial infarction (MI), myocardial ischemia reperfusion injuries (MIRI), and peripheral vascular diseases (PVD) [78]. Imaging techniques capable of visualizing and quantifying ROS provide invaluable insights into these disease processes, enabling early diagnosis, monitoring disease progression, and assessing the effectiveness of therapeutic interventions aimed at reducing oxidative stress [79].

Conventional imaging modalities like electrocardiography and echocardiography primarily focus on the heart’s electrical activity and structural/functional aspects, respectively [80]. Consequently, their ability to detect ROS overproduction is indirect and often lacks sensitivity and specificity [81]. While MRI and CT can utilize ROS-sensitive contrast agents, their specificity can be limited, and they may pose risks to certain patient populations [82]. These limitations highlight the need for advanced imaging techniques that can directly and specifically visualize ROS in vivo. A summary of ROS imaging in cardiovascular diseases are shown in Table 1. 

### 6.1. MI and MIRI

#### 6.1.1. Gadolinium (III)-Enhanced Cardiac MRI (Gd-CMR)

MI, a leading cause of morbidity and mortality globally, is intrinsically linked to an imbalance in redox homeostasis and subsequent ROS overproduction [83].While various imaging modalities have been explored to assess the extent of myocardial damage post-MI, Gd-CMR has emerged as a gold standard for evaluating infarct size, characterizing myocardial viability, and predicting patient outcomes [84].

The utility of Gd-CMR stems from the ability of GBCAs to distribute differentially within the myocardium [85]. In the setting of MI, necrotic tissue, characterized by disrupted cell membranes and increased extracellular space, allows for a greater GBCA accumulation compared to healthy tissue [86]. This differential uptake results in the enhanced contrast on T1-weighted images, enabling the clear demarcation of the infarct zone [87]. Importantly, the underlying pathophysiology of MI, particularly during the reperfusion phase, involves an overproduction of ROS [88]. Although Gd-CMR does not directly visualize ROS, the extent of late gadolinium enhancement on delayed images correlates with the severity of myocardial injury, which is partly mediated by ROS-induced damage [89].

#### 6.1.2. Gadolinium Ion-Integrated Cerium Oxide Nanoparticles (Gd-CeNPs)

Gd-CMR has become an invaluable tool for evaluating MI, but its uses are sometimes limited by concerns regarding gadolinium deposition and potential renal toxicity [90]. In this context, the emergence of Gd-CeNPs presents a promising avenue for advancing cardiac MRI in MI [91].

#### 6.1.3. Cerium Oxide Nanoparticles (CeNPs)

CeNPs are known for their intrinsic antioxidant properties, attributed to their ability to switch between Ce^3+^ and Ce^4+^ oxidation states, mimicking the activity of SOD and catalase enzymes [92]. Studies have shown that CeNPs can effectively scavenge ROS, including superoxide and H_2_O_2_, due to their ability to switch between Ce^3+^ and Ce^4+^ states. The Ce^3+^ state is associated with SOD-mimetic activity, while the Ce^4+^ state is associated with catalase-mimetic activity [92]. The reversible switching between Ce^3+^ and Ce^4+^ oxidation states allows CeNPs to act as regenerative antioxidants, effectively neutralizing multiple types of ROS [93]. The combined effects of the greater representation of Ce^3+^ at the nanocrystal surface and total molar surface area of 2 nm particles provide the maximum possible number of sites for the SOD and catalase mimetic reactions to occur compared to other nanostructures or sizes, and cerium oxide nanocrystals around 2 nm appear to be close to an optimum size for enzyme-mimetic activity [94]. By integrating gadolinium within CeNPs, researchers aim to create a theragnostic agent capable of both imaging MI and mitigating ROS-induced damage [95].

#### 6.1.4. ICG PEG-Ag2S Nanoprobe in PAI

ICG is a near-infrared fluorescent dye that has gained attention for its potential in imaging ROS-related tissue damage [96]. In the context of MI and MIRI, ICG’s ability to bind to damaged cell membranes and accumulate in areas of increased vascular permeability allows for the visualization of the infarct zone and regions of inflammation [97]. This can aid in assessing the extent of myocardial injury and monitoring the effectiveness of therapeutic interventions [98].

The integration of ICG with nanotechnology has further expanded its applications in imaging and treating cardiovascular diseases. ICG-loaded nanoparticles can be targeted to specific sites of injury, allowing for enhanced imaging sensitivity and localized drug delivery [99]. For instance, Wu developed an ICG@PEG-Ag2S nanoprobe for atherosclerosis targeting and imaging, demonstrating its ability to accumulate in atherosclerotic plaques and provide high-contrast photoacoustic imaging [100].

#### 6.1.5. PEG and Luminol-Conjugated Chlorin e6 (Ce6) Nanoprobes in Fluorescence Imaging

Fluorescent nanoprobes have emerged as powerful tools for imaging MI and the subsequent MIRI [101]. These nanoprobes, designed to respond to the elevated levels of ROS associated with these conditions, offer high specificity and real-time monitoring capabilities [102]. Ziegler et al. developed a self-assembled fluorescent nanoprobe for both the imaging and therapy of MIRI [101]. This innovative probe, composed of an amphiphilic copolymer incorporating PEG and luminol-conjugated Ce6, targets injured myocardium due to the local increase in ROS [103]. The unique design enables the nanoprobe to self-assemble into nanoparticles, facilitating its specific accumulation at the site of injury [104]. This targeted approach not only allows for the precise visualization of the ischemic/reperfused myocardium, but also opens avenues for localized drug delivery and therapy [105].

The ability to detect and monitor ROS in real-time is a key advantage of fluorescent nanoprobes [106]. A nanoprobe with high specificity for the ischemic/reperfused heart tissue and with a prolonged signal duration enables researchers to track the dynamics of oxidative stress and evaluate the efficacy of therapeutic interventions over time [101].

Ziegler et al.’s development of Ce6-luminol-PEG (CLP) nanoparticles exemplifies another innovative approach to ROS imaging [107]. Amphiphilic copolymer was generated by sequentially conjugating luminol and PEG with Ce6 and defined as CLP [107]. These nanoparticles utilize aggregation-induced fluorescence quenching, where the fluorescence of Ce6 is initially attenuated due to aggregation [108]. The presence of ROS in the damaged myocardium restores the fluorescence intensity of Ce6, allowing for the real-time detection and monitoring of the injured cardiac areas [109].

The dual functionality of fluorescent nanoprobes (in both imaging and in therapy) makes them an attractive material for the development of theragnostic agents [110]. By combining diagnostic capabilities with targeted drug delivery, these nanoprobes hold the potential to revolutionize the treatment of MI and MIRI [111]. They can be engineered to carry therapeutic payloads, such as antioxidants or anti-inflammatory agents, which are released in response to ROS, enabling localized treatment at the site of injury [112].

#### 6.1.6. Contrast-Enhanced Ultrasound (CEU) Molecular Imaging on MIRI

CEU molecular imaging uses the selective targeting and detection of encapsulated microbubbles (MBs) or other acoustically active agents that bind to molecules [113]. After intravenous injection of the agent, CEU images are obtained within minutes [114]. CEU molecular imaging techniques have been explored to study atherosclerosis, MIRI, angiogenesis,, and thrombus formation [111]. MBs have also been used to target activated endothelium using ligands for the endothelial selectin family of adhesion molecules that are expressed rapidly after ischemia, or by including phosphatidylserine in the shell of MBs [115]. This technique has been demonstrated in both murine and non-human primate models of MI [115].

N,N’-di-sec-butyl-N,N’-dinitroso-1,4-phenylenediamine (BNN6) has also been identified as an ultrasound-sensitive NO donor [101]. Xu et al. generated platelet membrane-coated nanoparticles that contain a PLGA core loaded with BNN6 (B-P@PLT), which allows for the controlled release of NO in response to US irradiation [101]. The platelet membrane coating of B-P@PLT enhances targeting to the damaged endothelium during MIRI [101]. The amount of NO released from B-P@PLT in the MIRI could be also controlled by adjusting the US parameters and irradiation time [101]. Xu et al. also evaluated the cardiomyocyte protection of B-P@PLT after US irradiation by assessing their effects on reducing the opening of the mitochondrial permeability transition pore, ROS, and the apoptosis of cardiomyocytes [101].

### 6.2. Peripheral Artery Disease (PAD)

#### 6.2.1. Oxidative Stress in PAD and Surrogate Biomarkers

PAD is one of the clinical presentations of atherosclerotic disease, characterized by impaired blood flow in the lower limbs, which causes intermittent claudication [116]. Subsequently, ischemia leads to infarction and tissue and limb loss in PAD. Oxidative stress plays a pathophysiological role in PAD [117]. Oxidative stress leads to the accumulation of post-translational bio-molecules (e.g., protein carbonylation or aldehyde/ketone adducts, nitration and sulfoxidation, DNA lesions such as 8-oxodG), further interfering with physiological redox capability [117,118].

#### 6.2.2. H_2_O_2_-Responsive Nanoparticles

PAD is under oxidative stress with a significantly high level of H_2_O_2_ [119]. A vanillyl alcohol-incorporated copolyoxalate, called PVAX, is designed to incorporate peroxalate ester linkages in its backbone and release bioactive vanillyl alcohol, while undergoing H_2_O_2_-consuming degradation [119]. Jung et al. developed H_2_O_2_-triggered CO_2_ bubbles generating CUR-PVAX nanoparticles as theragnostic agents for ischemic injuries [120]. In a hindlimb ischemia injury model in a mouse, CUR-PVAX nanoparticles showed a significant increase in ultrasound contrast in the ischemic area, which is associated with a high concentration of H_2_O_2_ [119].

Another group of H_2_O_2_-responsive nanoparticles are second-generation microbubble contrast agents, such as SonoVue^®^ (Bracco, Milan, Italy), Definity^®^ (Lantheus, MA, USA), and Sonazid^®^ (Daiichi Sankyo, Tokyo, Japan) [121]. However, these ultrasound contrast agents are limited in their ability to detect changes in vascular structure, such as in inflammatory diseases, PAD, or musculoskeletal tumors [122].

#### 6.2.3. Ultrasound Imaging-Guided Sonodynamic Therapy (SDT)

SDT is an non-invasive treatment that uses ultrasound to locally stimulate sonosensitizers to exert biological effects [123]. The stimulated sonosensitizers then produce ROS, which alters the cellular fate or function [124]. Jiang et al. showed that using SDT in combination with atorvastatin was more effective than using atorvastatin alone in PAD patients [123]. Additionally, SDT has good long-term efficacy. Yao et al. showed that SDT treatment for one month resulted in a significant reduction in neovascularization and arterial inflammation in a patient with PAD, which was an equivalent outcome compared to 3 months of intensive statin therapy [125,126].

### 6.3. Atherosclerosis

#### 6.3.1. Chitosan Nanococktail Containing Both Nanoceria and Superparamagnetic Iron Oxide Nanoparticles

The combination of nanoceria and iron-oxide nanoparticles is an interesting approach in the development of theragnostic platforms. Nanoceria has effective antioxidant properties and iron oxide nanoparticles enhance the MRI contrast [127,128,129,130,131,132]. When synthesized together in a theragnostic nanococktail, these materials offer a dual-functional platform, consisting of therapeutic action through ROS scavenging and diagnostic imaging via MRI contrast enhancement [133]. Both chitosan-coated iron oxide ceria oxide nanoparticles (Chit-IOCO) and chitosan-tripolyphosphate-coated iron oxide ceria oxide nanoparticles (Chit-TPP-IOCO) effectively reduced the ROS level of the lipopolysaccharide-stimulated macrophages. Chit-IOCO was less toxic to the cells and exhibited higher MRI relaxivity than Chi-TPP-IOCO (308 and 150 mM–1 s–1, respectively), indicating that Chi-IOCO was more effective than Chit-TPP-IOCO as an MRI contrast agent in macrophages [133].

#### 6.3.2. Microbubble CEU Molecular Imaging

Ultrasound molecular imaging offers a non-invasive and highly sensitive method to assess endothelial pro-inflammatory changes. It leverages the use of MBs as contrast agents to detect specific biomarkers within the vascular lumen [114,134]. For the detection of aggressive atherogenesis, CEU molecular imaging of adhesion molecules, such as selectins, intercellular-adhesion molecule-1, has been applied in murine models of disease [135]. These molecules are involved in the recruitment of innate immune cells into plaque. Hence, they have been found to provide one of the earliest markers for detecting the onset of aggressive atherogenesis [136]. Carotid CEU molecular imaging of P-selectin and the vascular cell adhesion molecule-1 in nonhuman primates has been shown to be highly effective in identifying early endothelial activation and prothrombotic vascular events, even before structural changes become apparent [111].

## 7. Applications of ROS-Targeted Imaging in Neurovascular and Neurodegenerative Diseases

### 7.1. Stroke

A summary of ROS imaging in non-cardiovascular diseases is shown in Table 2.

Stroke is the most common neurovascular disease, and one of the leading causes of morbidity and mortality in adults. ROS plays a critical role in stroke, resulting in apoptosis, disruption of the blood–brain barrier (BBB), inflammation, edema formation, autophagy, and other pathophysiological events [137]. One of the commonly used imaging modalities in stroke is MRI and MR angiography (MRA). Researchers are exploring and developing new MR techniques to improve the scavenging ROS that cause ischemia or reperfusion injury.

In the study by Du et al. [138], iron–gallic acid coordination polymer nanodots (Fe-GA CPNs) were developed to enhance medical imaging techniques, especially for ischemic stroke and its therapy. Fe-GA CPNs are engineered to serve a dual role in both therapeutic applications and as contrast agents for MRI. Since these nanodots possess magnetic properties, they allow them to enhance imaging quality, facilitating better diagnosis and monitoring of ischemic stroke. Also, Fe-Ga CPNs are designed to provide antioxidative properties that protect neurons from oxidative stress and apoptosis during ischemic events. These nanodots can also stimulate key neuroprotective signaling pathways such as inhibiting apoptosis through the restoration of protein kinase B, activating the nuclear factor erythroid 2-related factor 2, or activating heme oxygenase-1 pathways, which are associated with reduced apoptosis and improved cellular resilience against oxidative stress. Another aspect of biocompatibility and low toxicity makes these nanodots suitable for clinical applications in treating ischemic stroke, highlighting the potential of novel nanodots that can be utilized in positron emission tomography (PET)/MR imaging-guided ischemic stroke therapy.

Hyperpolarized ^13^C-MRI is another novel synthesized compound for detecting ROS in vivo [139]. By hyperpolarizing 5,5-dimethyl-1-pyrroline-N-oxide (DMPO) using dynamic nuclear polarization (DNP), it significantly increases the polarization of the nuclear spins of the ^13^C atoms. This technique enhances the NMR signal by more than 10,000-fold, allowing for lower compound concentrations in in vivo detection. Since DMPO is non-toxic at high bolus concentrations, it can be suitable for in vivo administration in animal models of ischemic stroke.

Detection of the BBB permeable MRI contrast agent, methoxycarbonyl-2,2,5,5-tetramethylpyrrolidine-1-oxyl (MC-P), represents a promising advancement in neuroimaging [140]. MC-P is a nitrogen agent that can provide insights into the redox status of brain tissues. Since ROS and compromised antioxidant defenses are implicated in various brain disorders, MC-P’s ability to participate in redox reactions allows for assessing oxidative stress in conditions, such as stroke and neurodegenerative diseases. The study demonstrated that MC-P significantly increases MRI signal intensity in brain regions, with up to 50% enhancements after injection. This improved sensitivity can lead to the better visualization of brain pathology and the more accurate diagnosis of neurological conditions. Furthermore, beyond imaging, MC-P’s antioxidant properties may offer protective effects against oxidative damage in brain tissues. This dual role as both a contrast agent and a potential therapeutic agent could be beneficial in managing oxidative stress-related brain disorders.

### 7.2. Alzheimer’s Disease (AD)

AD is the most common neurodegenerative disorder that is accompanied with cognitive decline and memory impairment leading to dementia. It is characterized by the accumulation of amyloid beta (Aβ) and neurofibrillary tangles due to the abnormal accumulation of phosphorylated tau proteins in the neuron [141]. In AD, the accumulation of heme in senile plaques has been linked to increased oxidative stress. Free heme can act as a pro-oxidant. If heme interacts with Aβ peptides, it can increase ROS production, leading to formation of neurofibrillary tangles and plaques [142]. Since the Raman spectra provide specific markers for different types of hemes present in the plaques, markers can help in identifying the types of hemoproteins, such as type-b or type-c, accumulating in plaques of AD. Therefore, if one can detect specific heme types in plaques through Raman imaging, it could lead to new diagnostic markers for AD. Also, it may broaden therapeutic strategies aimed at mitigating oxidative stress or targeting heme accumulation in the brain.

The H_2_O_2_-responsive fluorescent probe, specifically Aβ-targeted, enables the real-time detection and monitoring of H_2_O_2_ levels induced by Aβ aggregates in both cellular and animal models of AD [143]. Since H_2_O_2_ is associated with Aβ aggregation and induces neurotoxicity, detecting H_2_O_2_ levels allows researchers to investigate the relationship between oxidative stress and AD pathology, potentially identifying H_2_O_2_ as an early diagnostic biomarker [143]. Furthermore, fluorescent probes with NIR emission are widely used for disease diagnosis and therapeutic purposes, because of their properties such as low light scattering, deep tissue penetration, minimal photodamage, and low autofluorescence [144]. To enhance theragnostic potential of NIR fluorescence imaging, newly developed naphthylamine-based cyanine probes are introduced with promising features, such as low cytotoxicity, high fluorescence stability, better BBB penetration, and higher selectivity for Aβ oligomers and monomers.

### 7.3. Parkinson’s Disease (PD)

PD is a progressive neurodegenerative disease caused by diminished neurotransmitter level, increased oxidative stress, mitochondrial dysfunction, and disturbed protein homeostasis [145]. PD is accompanied with various motor symptoms, such as resting tremors, bradykinesia, muscle rigidity, and postural instability due to loss of dopaminergic neurons. NUU-1, a novel fluorescent probe, can be used for ROS fluorescence imaging to detect hypochlorous acid (HClO) in PD [146]. HClO is one kind of ROS, and the accumulation of ROS has a big impact on PD pathophysiology. Due to its high sensitivity and selectivity of HClO, NUU-1 was used to image basal HClO levels in mouse brain tissues, successfully distinguishing between PD-affected and normal tissues. This indicates the potential role of HClO in PD pathogenesis and the role of fluorescence imaging in the diagnosis of PD.

MRI can be also used as a guidance in the treatment of PD. MRI can provide close to the real-time imaging, allowing researchers to precisely target the area in the brain where gene therapy is needed. Thus, it is used in combination with ultrasound imaging as MRI-guided focused ultrasound by improving the delivery of gene therapy vectors across the BBB [147]. In the study, researchers focused on nuclear factor E2-related factor 2 (Nrf2) transfection because the Nrf2 gene is known as a neuroprotective gene counteracting ROS production by activating the Nrf2/ARE pathway. They used nano MBs as a carrier for the Nrf2 gene, since they can cross BBB and enter neurons so that they can target oxidative stress and neurodegeneration associated with PD. Also, since MRI can visualize nano MB’s location and monitor the opening BBB of ultrasound, it enhances the transfection efficacy. The conjugate of nano MBs and MRI-guided ultrasound is considered a non-invasive method, ensuring safety over surgical approaches and for repetitive treatments.

### 7.4. Multiple Sclerosis (MS)

MS is a progressive neurodegenerative disease with polyphasic inflammation caused by multifocal demyelination [148]. In active MS, ferrous iron ions accumulate extracellularly and uptake into microglia, which generates ROS [149]. For MS imaging, MRI is most commonly used for monitoring perivenular inflammation and enhancing plaques. Even though iron is involved in healthy brain functions, its excessive level leads to neurodegenerative diseases [150]. Since iron accumulation in deep grey matter nuclei may induce inflammation and neuronal damage due to oxidative stress, Gadolinium-enhanced MRI is currently used in clinics by enhancing T1-weighted images that show BBB damage and active lesions. However, it only offers a small time frame for detecting active lesions, so it can’t distinguish multiple pathological features. Therefore, it is better to combine quantitative susceptibility mapping (QSM) to gadolinium-enhanced MRI since it provides a more accurate measure of iron content through differentiating between active and chronic lesions.

Since most MRI methods are not much sensitive to oxidative stress at cellular level, QUEnch-assiSTed (QUEST) MRI can monitor overall redox state instead of monitoring a single antioxidant or free radical [151]. It measures the relaxation time (T1) changes associated with the presence of paramagnetic free radicals during oxidative stress. During the scan, an antioxidant treatment is administered to quench the free radicals, which helps us to see the impact of ROS on the observed changes in relaxation rates (R1).

## 8. Applications of ROS-Targeted Imaging in Other Disease Conditions

### 8.1. Dermatology

In vivo confocal Raman spectroscopy is a powerful tool for understanding skin aging. It provides detailed insights into the molecular and structural changes occurring in the skin’s extracellular matrix (ECM) due to both intrinsic and extrinsic aging processes [152]. In particular, UV exposure leads to ROS production, which activates matrix metalloproteinases (MMPs) that degrade collagen and elastin in the dermis. Raman spectroscopy can detect that these outcomes resulted from oxidative damage. Raman spectroscopy allows identifying specific molecular changes in the skin that are associated with oxidative stress, including the formation of advanced glycation end products (AGEs) and the degradation of collagen, by linking AGE markers producing more ROS.

MRI can be also used for monitoring ROS levels in skin aging. Synthesized chiral manganese oxide (MnO_2_) nanoparticles used with dual signals of circular dichroism and MRI showed super sensitivity and high specificity for ROS detection, both in vivo and in living cells [153]. Since manganese has multiple valences, chiral MnO_2_ NPs can be used for ROS analysis, especially with H_2_O_2_. Chiral MnO_2_ NPs can also effectively eliminate H_2_O_2_ because H_2_O_2_ is decomposed to O_2_ and H_2_O after reacting with chiral MnO_2_ NPs. Furthermore, chiral MnO_2_ NPs can inhibit the expression of aging-associated biomarkers, such as interleukin (IL)-6, nuclear lamina protein B1, IL1*β*, and p21, confirming that all aging-related biomarkers returned to almost normal levels after treating with chiral MnO_2_ NPs. These were also examined on mouse skin, and showed increased activity of antioxidant enzymes, such as catalase, glutathione (GSH), and GSH peroxidase after treatment with chiral MnO_2_ NPs [153]. Therefore, chiral MnO_2_ NPs enable the real-time monitoring of ROS via targeted ROS scavenging. Many of the metal-based agents have been developed for the catalytic redox ability and high tensile strength but clinical translation has been limited due to their toxic potentials.

### 8.2. Cancer

ROS plays a significant role in tumor cells developing as a cancer. Inside tumor cells, an elevated level of ROS causes damage to DNA, proteins, and lipids, which promotes genetic instability [154]. Also, ROS acts as a signaling molecule between cancer cells by promoting tumorous cell growth and metastasis, and making them resistant to apoptosis. Therefore, imaging ROS is crucial in monitoring the tumor microenvironment and probing mechanisms of cancer.

To enhance PAI effectiveness, researchers designed activatable ABTS@MIL-100/PVP nanoreactors (AMP NRs) for in vivo tumor theragnostic agents [155]. Since PAI signals of AMP NRs were activated by H_2_O_2_ and increased with the concentration of H_2_O_2_, it showed remarkable selectivity for tumor-specific imaging and therapy [155]. Also, when AMP NRs were injected in mice with tumors, the PAI signals increased over time in the tumor. On the other hand, PAI signals were negligible in the nontumor subcutaneous tissue of other mice injected with AMP NRs. Moreover, the tumor-specific PAI ability of AMP NRs has been demonstrated in the study, showing that it can also image tumors at early stages. The PAI intensity dramatically increased with the tumor whose volume was nearly 20 mm^3^, showing that AMP NRs have the potential to detect tumors at very early stages.

Fluorescence imaging can be also utilized in surgery and radiative treatment. VGd@ICG-FA nanoprobe is synthesized to increase the sensitivity of radiotherapy in breast-conserving surgery, designed as a virus-like silicon dioxide nanoprobe with Gd coating and folic acid, coated with a layer of tetra sulfide linkages, and loaded with ICG [156]. The VGd@ICG-FA nanoprobe significantly increases the tumor-to-background ratio in NIR-II fluorescence imaging, allowing for the more precise visualization of tumor boundaries during surgery. This helps achieve negative surgical margins and accurately delineate tumor locations, which is crucial for effective surgical resection. Also, through NIR-II fluorescence imaging, the VGd@ICG-FA nanoprobe allows real-time monitoring, such as changes in ROS level, so clinicians can monitor the effectiveness of therapy and the trend of tumor behavior. Moreover, this nanoprobe can induce ferroptosis by depleting glutathione levels and increasing ROS levels in the cancer cell when gadolinium is exposed to radiation. Then the accumulation of ROS leads to cell death. Therefore, there is a potential for fluorescence imaging to be utilized for both diagnostic and therapeutic purposes in cancer.

### 8.3. Acute Kidney Injury (AKI)

AKI is one of the common ROS-related renal diseases in which excessive ROS from renal infiltrating or endogenous cells damages kidneys [157]. Near-infrared window II (NIR-II) fluorescent probing has several significant advantages for monitoring ROS in the kidney [158]. ICG is utilized as part of a fluorescent sensor system with NIR-II, allowing deeper penetration into biological tissues than visible light and reducing autofluorescence from biological tissues. Also, NIR-II probes can be designed to respond to ROS, enabling both in vivo imaging and in vitro urine analysis for comprehensive monitoring. In the study, the GNP-KTP5-ICG sensor utilizes a mechanism where ICG fluorescence is quenched when conjugated to gold nanoparticles (GNP). In the presence of ROS, such as H_2_O_2_, the bond between the ICG and GNP is disrupted, leading to the recovery of ICG fluorescence. This also can work as a dual-mode early detection strategy in which the ICG-KTP5 remains in the kidney and provides in vivo imaging of kidney injury, while detached GNP can be excreted into the urine, allowing for in vitro analysis. Since continuous monitoring of ROS levels can provide insights into the progression of kidney dysfunction over time, NIR-II fluorescent probes represent a promising advancement in imaging and diagnostic approaches.

The endogenous biopolymers can also be utilized for ROS scavenging in the kidney. Since melanin has profound characteristics, such as near-infrared absorbance, strong chelating capability, and natural antioxidative capability, it can be utilized for bimodal imaging as a nanoparticle [159]. An ultrasmall polyethylene glycol-incorporated (PEGylated) Mn^2+^-chelated melanin (MMPP) nanoparticle is synthesized to enhance the imaging quality of bimodal imaging modality, which is integrated with PET and MRI. MMPP nanoparticles exhibit excellent T1 relaxivity (r1) in MR imaging, and it is significantly higher than that of conventional Gd-based contrast agents. This high relaxivity enhances the contrast in MR images, making it easier to visualize the kidneys and assess the therapeutic effects. Also, MMPP can be radiolabeled with Zirconium-89 (^89^Zr) through a chelator-free method, enabling PET imaging to track the nanoparticles’ accumulation in target tissues. Moreover, since MMPP shows a strong antioxidative response by significantly reducing oxidative stress and improving kidney function indicators in a model of AKI in mice, as a potent antioxidant defense.

### 8.4. Inflammatory Rheumatologic Diseases

Rheumatoid arthritis is a chronic inflammatory joint disease that can lead to significant disability and mortality [160]. To enhance the ROS scavenging ability of PAI, M@P-siRNAs^T/I^ incorporated Prussian blue nanoparticles (PBNPs) and small interfering RNAs (siRNAs) within macrophage membrane vesicles (MMVs) [161]. Since PBNPs have intrinsic enzyme-like abilities, such as peroxidase, catalase, and superoxide dismutase functions, they can promote the dismutation of H_2_O_2_. Also, the ability of NIR absorbance of PBNPs can lead to real-time monitoring through PAI, it allows researchers to monitor the distribution and accumulation of the nanoparticles in inflamed tissues, correlating the therapeutic effects with the observed PA signals. Additionally, siRNAs^T/I^ have specific gene silencing capability, suppressing the expression of proinflammatory cytokines, and this can further contribute to lowering ROS production in the inflamed tissues. As PBNPs and siRNAs^T/I^ are incorporated in MMVs, stability and biocompatibility are improved so that targeting of inflamed tissues is enhanced, ensuring that the ROS scavenging activity of PBNPs is concentrated where it is most needed.

Cu-coordinated polyphthalocyanine-based artificial peroxisomes (CuAP) are developed for ROS scavenging with PAI [162]. Since CuAPs exhibit strong optical absorption in the near-infrared region around 750 nm, they serve as effective contrast agents for PAI, allowing the real-time monitoring of the distribution and accumulation of CuAP in inflamed joints during arthroscopic treatment. Also, CuAP tends to accumulate in an inflamed area, so the intensity of the photoacoustic signal tends to be higher at that area, which has a higher concentration of CuAP. This allows for the precise monitoring of treatment. Furthermore, CuAP has catalytic activity, and recruits the macrophages that could scavenge ROS. The concept of artificial peroxisome is innovative, but it needs thorough toxicity and safety studies for human use.

## 9. Limitations of ROS Imaging

Recent advancements in ROS imaging have shown great promise, but despite their significant diagnostic and therapeutic potentials, their clinical application remains limited. Several challenges hinder their widespread use. The rapid generation and very short half-life of ROS makes real-time imaging challenging in a clinical setting. One potential solution is the real-time imaging of ROS accumulation using portable or implanted devices. In clinical practice, sonographic techniques can offer real-time imaging through hand-held probes, but a sonogram requires substantial user expertise. Research has also explored using contrast agents, such as MBs, for real-time ultrasound ROS imaging. Safety is another critical consideration for both the imaging modalities and contrast agents used. While materials targeting ROS overproduction are permissible in animal research and ex vivo studies without FDA approval, clinical research mandates such approval, limiting researchers’ options. In addition, we need specific and sensitive ROS-targeting materials for a specific form of ROS that could be safely used for imaging.

## 10. Conclusion and Future Perspectives

This review underscores the potential of ROS imaging as a valuable diagnostic tool for preventing and managing ROS-related conditions. Our review also highlights the inherent complexities and variabilities of these techniques. The combination of ROS-targeted imaging materials with various imaging modalities could further enhance these techniques’ development. Various optically based imaging and fluorescence-based contrasts have improved for better visibility and conspicuity for ROS and oxidative stress evaluation but remain constrained by the limited absorption of lighting and scattering of emitted light by the overlying tissue. Depth perception and surround anatomic details are helped by advanced imaging modalities, such as PET, US, CT, and MR, but each has own pros and cons. Depending on the disease and/or patient conditions, a suitable specific modality paired with a contrast agent might lead to its successful transition to clinical usage. Continued research in chemistry, materials science, engineering, and medicine, along with advancements in fusion biotechnology, is essential to realize the unique potential of developing clinically promising ROS-imaging agents and techniques.

## Figures and Tables

**Table 1:** ROS imaging in cardiovascular diseases.

Disease (System)	Imaging modalities	Application and Therapeutic Effect	Functionality
MI	MRI	Gadolinium (III)-enhanced cardiac MRI, extent of late gadolinium enhancement on delayed images correlates with the severity of myocardial injury	ROS scavenging Real-time Reduce infarct size
		Gd-CeNPs, mimicking the activity of superoxide dismutase and catalase enzymes	MI imaging, mitigating ROS-induced damage
	Photoacoustic imaging	Indocyanine green (ICG), near-infrared fluorescent dye contrast	Visualizing infarct zone, assessing myocardial injury
MIRI	Fluorescence imaging	Fluorescent nanoprobe (PEG, luminol-conjugated Ce6)	Real-time ROS monitoring
	Ultrasound	Contrast-enhanced ultrasound (CEU) molecular imaging with B-P@PLT nanoparticles	Reducing ROS and cardiomyocytes
Atherosclerosis (Vascular)	MRI	Chitosan nanococktails containing nanoceria and superparamagnetic iron oxide nanoparticles	Effective ROS scavenging, MRI contrast
	Ultrasound	Microbubble CEU molecular imaging	Imaging of adhesion molecules ICAM-1, detecting prothrombic vascular events
		US-imaging guided sonodynamic therapy	Long-term efficacy, reduction in neovascularization and arterial inflammation
PVD (Vascular)	CT/MRI/PET	Oxidative stress surrogate biomarkers	ROS targeting
	IVUS/OCT	IVUS/OCT imaging modalities used to provide high resolution and differentiate various plaque components	High-resolution view, vessel wall visualization and plaque microstructure
	Ultrasound	CUR-PVAX nanoparticles PVO	H_2_O_2_-responsive, suppressing oxidative stress, suppressing proinflammatory TNF-a and IL-1b, and VEGF
		SonoVue microbubble contrast	High signal intensity, 3 h contrast enhancement time

**Table 2:** ROS imaging in non-cardiovascular diseases.

Disease (System)	Imaging Modalities	Application and Therapeutic Effect	Functionality
Stroke	MRI	Ultrasmall iron–gallic acid coordination polymer nanodots (Fe-GA CPNs) guided MRI Potent antioxidant ^13^C MRI with hyperpolarized DMPO BBB permeable nitroxide (MC-P) for MRI contrast agent Potential antioxidant	ROS scavenging Real-time imaging Reduce ROS and apoptosis In vivo free radical imaging ROS scavenging Real-time ROS monitoring Antioxidant property
Alzheimer’s Disease	Fluorescence imaging	H_2_O_2_-responsive fluorescent probe with near-infrared (NIR) emission	Real-time ROS detection and monitoring
Parkinson’s Disease	Fluorescence imaging	NUU-1 fluorescence imaging for HClO with high sensitivity and rapid response	HClO imaging Distinguish PD tissue from normal control
	MRI/Ultrasound	MRI-guided focused ultrasound delivery of microbubbles containing nuclear factor E2-related factor (Nrf2)	Targeting ROS production with Nrf2 gene Opening BBB with ultrasound MRI monitoring for transfection
Multiple Sclerosis	MRI	Quantitative susceptibility mapping (QSM) with T2 relaxation contrast in MRI QUEnch-assiSTed (QUEST) MRI	Iron-sensitive imaging
			ROS monitoring No contrast agent needed
Skin aging	Raman imaging	In vivo confocal Raman spectroscopy for AGE accumulation leading to ROS formation	ROS-induced damage detection ROS biomarker monitoring
Acute Kidney Injury	Fluorescence imaging	Indocyanine green NIR-II probe GNP-KTP5-ICG sensor	ROS imaging and monitoring Dual-mode early detection strategy
	PET/MRI	Mn^2+^-chelated melanin (MMPP) nanoparticle-integrated PET imaging/MRI Potent antioxidant	ROS scavenging Visualizing kidneys for therapeutic effect Strong antioxidative response of MMPP
Rheumatoid Arthritis	PAI	Prussian blue nanoparticles (PBNPs) and siRNAs incorporated nanoparticle (M@P-siRNAs^T/I^) guided near-infrared (NIR) PA imaging	ROS scavenging TNF-α and IL-6 suppression, lowering ROS production
	Fluorescence imaging	Cu-coordinated polyphthalocyanine-based artificial peroxisomes (CuAP) incorporated with ultrasound- and microbubble-guided fluorescence imaging	ROS scavenging Lowering number of osteoclasts Anti-inflammatory ability
Cancer	PAI	ABTS@MIL-100/PVP nanoreactors (AMP NRs) for in vivo PA imaging	H_2_O_2_ scavenging Remarkable selectivity for tumor-specific imaging
	Fluorescence imaging	VGd@ICG-FA nanoprobe NIR-II fluorescence imaging	Real-time ROS monitoring Potent radiotherapy sensitization
